# Thrombospondin-2 and LDH Are Putative Predictive Biomarkers for Treatment with Everolimus in Second-Line Metastatic Clear Cell Renal Cell Carcinoma (MARC-2 Study)

**DOI:** 10.3390/cancers13112594

**Published:** 2021-05-25

**Authors:** Philip Zeuschner, Sebastian Hölters, Michael Stöckle, Barbara Seliger, Anja Mueller, Hagen S. Bachmann, Viktor Grünwald, Daniel C. Christoph, Arnulf Stenzl, Marc-Oliver Grimm, Fabian Brüning, Peter J. Goebell, Marinela Augustin, Frederik Roos, Johanna Harde, Iris Benz-Rüd, Michael Staehler, Kerstin Junker

**Affiliations:** 1 Department of Urology and Pediatric Urology, Saarland University, Kirrberger Street, 66421 Homburg/Saar, Germany; Philip.zeuschner@uks.eu (P.Z.); sebastian.hoelters@t-online.de (S.H.); Michael.stoeckle@uks.eu (M.S.); 2 Institute of Medical Immunology, Martin Luther University Halle-Wittenberg, Universitätsplatz 10, 06108 Halle (Saale), Germany; barbara.seliger@uk-halle.de (B.S.); anja.mueller@uk-halle.de (A.M.); 3 Center for Biomedical Education and Research (ZBAF), Faculty of Health, Institute of Pharmacology and Toxicology, School of Medicine, Witten/Herdecke University, Stockumer Straße 10, 58453 Witten, Germany; Hagen.Bachmann@uni-wh.de; 4 Department of Hematology, Hemostasis, Oncology and Stem Cell Transplantation, Hannover Medical School, University Hospital, Carl-Neuberg-Strasse 1, 30625 Hannover, Germany; Viktor.Gruenwald@uk-essen.de; 5 Clinic for Internal Medicine (Tumor Research) and Clinic for Urology, West German Cancer Center, University Hospital Essen, Hufelandstrasse 55, 45147 Essen, Germany; 6 Department of Medical Oncology, University Hospital Essen, Hufelandstrasse 55, 45147 Essen, Germany; d.christoph@kem-med.com; 7 Department of Medical Oncology & Hematology, Evang. Kliniken Essen-Mitte, Evang. Huyssens-Stiftung Essen-Huttrop, Henricistrasse 92, 45136 Essen, Germany; 8 Department of Urology, University Hospital Tuebingen, Hoppe-Seyler-Strasse 3, 72076 Tübingen, Germany; arnulf.stenzl@med.uni-tuebingen.de; 9 Department of Urology, University Hospital Jena, Am Klinikum 1, 07747 Jena, Germany; marc-oliver.grimm@med.uni-jena.de; 10 Department of Urology and Pediatric Urology, University Hospital Giessen and Marburg GmbH, Philipps-University Marburg, Baldingerstrasse, 35033 Marburg, Germany; apofab@web.de; 11 Ambulatory Uro-Oncological Therapy Unit Erlangen (AURONTE), Department of Urology and Clinic for Haematology and Internistic Oncology, University Hospital Erlangen, Krankenhausstrasse 12, 91054 Erlangen, Germany; peter.goebell@uk-erlangen.de; 12 Department of Hematology and Oncology, Paracelsus Medical University, Prof.-Ernst-Nathan-Strasse 1, 90419 Nürnberg, Germany; marinela.augustin@klinikum-nuernberg.de; 13 Department of Urology and Paediatric Urology, University Medical Center Mainz, Langenbeckstraße 1, 55131 Mainz, Germany; Frederik.Roos@kgu.de; 14 Department of Urology, University Hospital Frankfurt, Theodor-Stern-Kai 7, 60590 Frankfurt, Germany; 15 Medical Department, iOMEDICO, Ellen-Gottlieb-Strasse 19, 70106 Freiburg im Breisgau, Germany; johanna.harde@iomedico.com (J.H.); Iris.Benz-Rued@iomedico.com (I.B.-R.); 16 Interdisciplinary Center of Renal Tumors, Department of Urology, Ludwig-Maximilians-University of Munich, Marchioninistrasse 15, 81377 Munich, Germany; Michael.Staehler@med.uni-muenchen.de

**Keywords:** biomarker, everolimus, metastatic renal cell carcinoma, second-line, phase IV

## Abstract

**Simple Summary:**

Treatment of metastatic renal cell carcinoma (mRCC) remains a challenge due to the lack of biomarkers indicating the optimal drug for each patient. This study analyzed blood samples of patients with predominant clear cell mRCC who were treated with the mTOR inhibitor everolimus after failure of one prior tumor therapy. In an exploratory approach, predictive blood biomarkers were searched. We found lower levels of the protein thrombospondin-2 (TSP-2) at the start of the therapy and higher lactate dehydrogenase (LDH) levels in serum two weeks after therapy initiation to be associated with therapy response. Of note, these blood biomarkers had a higher predictive value than baseline patient parameters or risk classifications. Polymorphisms in the mTOR gene appeared to be associated with therapy response, but were not significant. To conclude, it seems feasible to identify patients showing longtime responses to everolimus and possible to increase tumor therapy response rates based on biomarkers for individual therapy selection.

**Abstract:**

There is an unmet need for predictive biomarkers in metastatic renal cell carcinoma (mRCC) therapy. The phase IV MARC-2 trial searched for predictive blood biomarkers in patients with predominant clear cell mRCC who benefit from second-line treatment with everolimus. In an exploratory approach, potential biomarkers were assessed employing proteomics, ELISA, and polymorphism analyses. Lower levels of angiogenesis-related protein thrombospondin-2 (TSP-2) at baseline (≤665 parts per billion, ppb) identified therapy responders with longer median progression-free survival (PFS; ≤665 ppb at baseline: 6.9 months vs. 1.8, *p* = 0.005). Responders had higher lactate dehydrogenase (LDH) levels in serum two weeks after therapy initiation (>27.14 nmol/L), associated with a longer median PFS (3.8 months vs. 2.2, *p* = 0.013) and improved overall survival (OS; 31.0 months vs. 14.0 months, *p* < 0.001). Baseline TSP-2 levels had a stronger relation to PFS (HR 0.36, *p* = 0.008) than baseline patient parameters, including IMDC score. Increased serum LDH levels two weeks after therapy initiation were the best predictor for OS (HR 0.21, *p* < 0.001). mTOR polymorphisms appeared to be associated with therapy response but were not significant. Hence, we identified TSP-2 and LDH as promising predictive biomarkers for therapy response on everolimus after failure of one VEGF-targeted therapy in patients with clear cell mRCC.

## 1. Introduction

The introduction of targeted therapies, including tyrosine kinase inhibitors (TKI, such as sorafenib, sunitinib, pazopanib, axitinib, cabzoantinib, tivozanib); anti-VEGF antibodies, such as bevacizumab; and mammalian target of rapamycin (mTOR) inhibitors (everolimus, temsirolimus) has revolutionized the treatment of metastatic renal cell carcinoma (mRCC) in recent years [1,2,3]. More recently, immune checkpoint inhibitors (CIs), such as nivolumab and ipililumab, and their combination with targeted therapy agents (pembrolizumab/avelumab + axitinib) have further broadened the armamentarium of clinicians treating mRCC [4,5]. However, most studies concentrate on identifying new substances with higher response rates for more patients and therefore neglect the fact that non-responders might also profit from other or earlier substances. As a result, there is a significant lack of predictive biomarkers indicating the optimal drug for the individual patient in mRCC.

The MARC-2 study was a single-arm open-label phase IV study including patients with predominantly clear cell mRCC after exactly one prior VEGF-based therapy. Patients were treated with everolimus (Afinitor^®^), which was considered as the standard of care in second- or third-line therapy of clear cell mRCC after the failure of VEGF-targeted therapies at the time of the study initiation in 2011 [3,6]. During this era of TKI treatment, the only registered drugs in this setting besides everolimus were axitinib and sorafenib [7]. The clinical results and baseline parameters of the MARC-2 study have recently been presented and they have underlined the safe and effective profile of everolimus [7]. Patients aged ≥65 years (6-month progression-free survival (PFS) 54.4% (95% confidence interval (95% CI) 35.2–70.1) vs. 23.7% (95% CI 10.5–39.9)) and with a BMI >25 kg/m² (6-month PFS 51.4% (95% CI 34.7–65.7) vs. 18.2% (95% CI 5.7–36.3)) gained the most benefit [7].

However, the MARC-2 study also aimed to identify blood biomarkers, including angiogenesis-related proteins, metabolic molecules, and mTOR polymorphisms. Here, we present our findings on predictive biomarkers for everolimus in the second-line setting for the treatment of clear cell mRCC.

## 2. Materials and Methods

The MARC-2 trial, registered at ClinicalTrials.gov (NCT01266837), was conducted according to the declaration of Helsinki. The study protocol was reviewed by the independent ethics committee or the institutional review board for each center. Each patient provided written informed consent before the screening procedures were initiated. The study design, patient characteristics, and clinical outcomes of the prospective MARC-2 trial have been described elsewhere [7]. In brief, adult patients with predominant clear cell mRCC who had progressed during or after exactly one prior VEGF-TKI therapy were treated with 10 mg of everolimus orally until disease progression. The patients were enrolled between January 2011 and August 2015. As a primary outcome, the multi-center, single-arm study investigated the six-month PFS, while the identification of predictive biomarkers for therapy response served as a secondary outcome. Blood samples for biomarker assays were taken on the day of treatment initiation prior to the first everolimus application, on the 15th day of the first treatment cycle (C1D1, C1D15), and on every first day of the subsequent treatment cycles, each lasting 28 days.

In total, 63 patients were enrolled in the study and received at least one dose of everolimus; they were defined as the full analysis set (FAS). The per protocol (PP) group consisted of 48 patients out of the FAS who fulfilled all inclusion but no exclusion criteria. Patients in the PP group had a relative dose intensity of at least 50% in the first two treatment cycles as well as a tumor response evaluation prior to day 182, or progressed, discontinued treatment due to an adverse event, or died before the minimum exposure requirement [7]. Treatment efficacy was assessed in the pre-specified subgroups, as well as patient gender, age at date of informed consent (≥65 vs. <65years), body mass index (BMI) at screening (>25 vs. ≤25 kg/m²), and Eastern Co-operative Oncology Group (ECOG) performance at baseline (≥1 vs. 0) [7]. The patients were also classified according to the international metastatic renal cell carcinoma (RCC) database consortium (IMDC) risk criteria [8].

A total of 55 angiogenesis-related proteins were screened by antibody arrays (Proteome ProfilerTM Array, Human Angiogenesis Array Kit; Catalog Number ARY007; R&D Systems Europe, Ltd., Abingdon, UK) in 12 patients (eight responders, four non-responders) at C1D1 and C1D15. Potential good candidates for biomarkers were validated by ELISA (Quantikine^®^ ELISA; R&D Systems Europe, Ltd., Abingdon, UK) according to the manufacturer’s protocols in 41 patients at C1D1 and 36 patients at C1D15. The concentration of each protein was normalized to the total plasma protein concentration and defined as parts per billion (ppb). The expression of the metabolic molecules lactate dehydrogenase (LDH), enolase-1 (ENO1), and superoxide dismutase 2 (SOD2) was measured for 53 patients by ELISA technology (Abcam, Berlin, Germany). In addition, the polymorphisms mTOR3162/rs2295080 and mTOR3099/rs2295079 in the mTOR promoter region and mTOR8600/rs2356 and mTOR8167/rs12139042 in the mTOR 3′ untranslated region were analyzed for 54 patients. Genotyping was performed as previously described [9].

Statistical analyses were carried out both in the FAS and PP cohort; results are given in the FAS, if not indicated otherwise. In order to find biomarkers with a high discriminative power for long therapy responders, long responders were defined as patients with stable disease (SD) or partial response (PR) at day 182 or later and compared with early non-responders who had progressive disease (PD) at day 56. Categorical variables are reported as frequencies and proportions, and continuous data as the median and range. Fisher’s exact, Mann–Whitney U, and Kruskal–Wallis tests were applied to compare the groups. The cut-offs and the c-index for prediction were calculated by receiver operating characteristic (ROC) analysis. The PFS and overall survival (OS), including 95% confidence intervals, were estimated with the Kaplan–Meier method and compared with log rank tests. To compare the predictive impact of the identified biomarkers (TSP-2 C1D1: >665 vs. ≤665 ppb; TSP-2 C1D15: >635 vs. ≤635 ppb or LDH C1D15: >27.14 vs. ≤27.14 nmol/L) with IMDC risk groups (favorable + intermediate vs. poor) and pre-specified patient cohorts (age group: ≥65 vs. <65 years; gender: Male vs. female; BMI: >25 vs. ≤25 kg/m²), univariate and multiple Cox regressions were performed for the OS and PFS. The independent binary variables were only included in the multiple regression if the respective effect was significant in the univariate analysis; Forward Wald selection was applied. To assess the impact of a potential lead time bias, the Kaplan–Meier and Cox regression analyses were conducted again in a separate dataset, with the landmark set to C1D15, as previously described [10]. In the event of missing data, cases were excluded. The statistical analyses were performed with SPSS version 25 (IBM, Armonk, NY, USA). All the tests were two-sided, and *p*-values < 0.05 were considered significant.

## 3. Results

### 3.1. Biomarker Expression

In order to screen for biomarker candidates associated with angiogenesis, antibody arrays were performed in a test cohort comprising eight responders and four non-responders. Platelet-derived growth factor-AA (PDGF-AA), angiopoietin-1 (Ang1), and thrombospondin-2 (TSP-2) had a trend to be higher in early non-responders than in long responders (see Supplementary Material Figure S1). Moreover, blood levels appeared to increase in non-responders but to decrease in responders from C1D1 to C1D15. However, these differences were not statistically significant.

**Figure 1:**
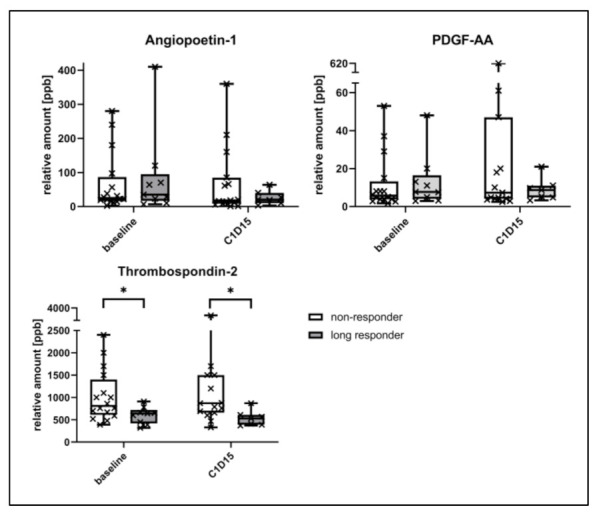
Comparison of angiogenesis-related blood proteins between long responders with early non-responders, measured by ELISA quantification. * *p*-value < 0.05; PDGF: Platelet-derived growth factor-AA; ppb: Parts per billion.

These three candidate biomarkers were validated in a larger cohort of 41 patients by ELISA. TSP-2 was again lower for long responders compared to non-responders at C1D1 (median 640 ppb (range 320–910) vs. 810 ppb (390–2400), *p* = 0.032) and C1D15 (530 ppb (370–870) vs. 870 ppb (330–3400), *p* = 0.011; see Figure 1). By conducting ROC analyses, 665 ppb (c-index 0.76 (95% CI 0.57–0.95), *p* = 0.034) and 635 ppb (0.83 (95% CI 0.66–1.0), *p* = 0.014) were calculated as the best cutoffs for TSP-2 at C1D1 and C1D15 to differentiate long-term responders from early progressors (see Supplementary Material Figure S2). The ratio of TSP-2 at C1D15 to C1D1 was not statistically different. For Ang1 and PDGF-AA, neither the ratio nor the comparison of long vs. non-responders was significant in the validation cohort.

**Figure 2:**
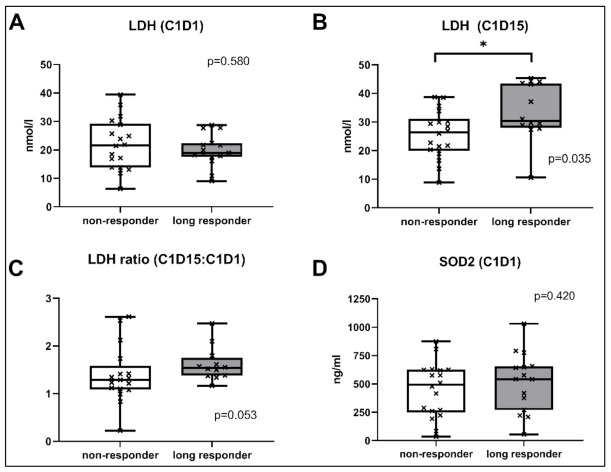
Comparison of the LDH levels at C1D1 (**A**), C1D15 (**B**), the LDH ratio (**C**) or SOD2 levels at C1D1 (**D**) between long responders and early non-responders. * *p*-value < 0.05; LDH: Lactate dehydrogenase, SOD2: Superoxide dismutase 2.

The metabolic markers LDH, ENO1, and SOD2 were selected to determine their suitability as predictive biomarkers. The LDH at C1D15 was significantly higher in long-term responders compared to early non-responders (median 30.36 nmol/L (range 10.57–45.36) vs. 26.46 nmol/L (8.82–38.67), *p* = 0.035; see Figure 2), as defined by ELISA. The value of 27.14 nmol/L was calculated as the optimal cut-off (c-index 0.73 (95% CI 0.54–0.92), *p* = 0.034; see Figure S2) via ROC analysis. The ratio of LDH (C1D15 to C1D1) also had a trend to be higher in responders (median 1.54 (range 1.16–2.47) vs. 1.29 (0.22–2.61), *p* = 0.053), but this was not statistically significant. Neither the LDH at other time points nor the ENO1 and SOD2 levels were different between responders and non-responders.

**Figure 3:**
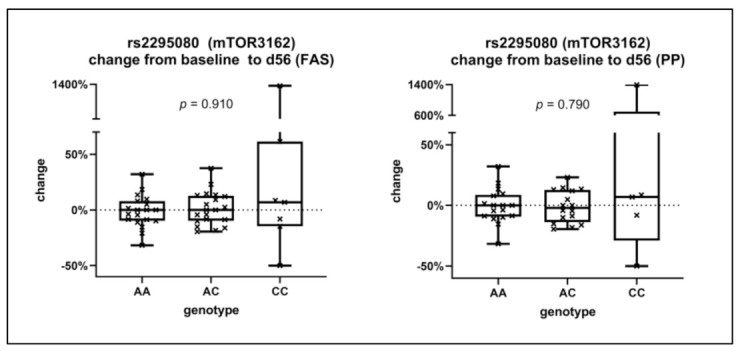
Relative change of the size of the target lesion on day 56 compared to baseline, stratified by the genotype of the rs2295080 (mTOR3162) polymorphism. The results are given in the full analysis set (FAS, left) and the per protocol analysis (PP, right).

The minor allele frequency of the mTOR polymorphism pair rs2295080/rs2295079 was 0.370 and 0.065 for rs2536/rs12139042 (see Supplementary Material Table S1 for genotype distributions). The genotypes of all polymorphisms were in the Hardy–Weinberg equilibrium, and their corresponding *p*-values were 0.35 (rs2295080/rs2295079) and 0.61 (rs2536/rs12139042). When stratifying the radiological change in the size of the target lesion on day 56 (compared to baseline) by the genotype of the rs2295080 polymorphism, carriers of the homozygous CC genotype had a tendency towards a stronger increase in the target lesion (see Figure 3). Accordingly, the proportion of patients with progressive disease at day 56 was higher for CC carriers (43%) compared to AC (29%) and AA carriers (35%, see Supplementary Material Table S2). However, these differences did not reach statistical significance.

### 3.2. Oncological Outcome, Stratified by Patient Groups and Biomarkers

Within the FAS, all 63 patients had a 6-month PFS of 39.3% (95% CI 27.0–51.3), with a median PFS of 3.8 months (95% CI 3.2–6.2) and an OS of 16.8 months (95% CI 14.3–24.3, see Table 1). Patients at favorable risk (median OS 20.4 months) had a longer OS than the intermediate and poor risk group (18.9 months (95% CI 9.9–27.9) vs. 6.8 (95% CI 3.8–9.6), *p* = 0.052, see Supplementary Material Figure S3), but this difference did not reach statistical significance. Accordingly, the 6-month PFS rate was higher in the favorable risk group (47.6% (95% CI 7.5–80.8) than for the intermediate and poor (44.6% (95% CI 28.9–59.2) vs. 9.1% (95% CI 0.5–33.3), *p* = 0.057) risk groups, respectively. However, when stratifying favorable and intermediate vs. poor risk, the PFS (5.7 months (95% CI 2.4–9.0) vs. 3.6 (95% CI 1.6 vs. 5.5), *p* = 0.025) and OS (20.4 months (95% CI 11.1–29.7) vs. 6.8 (95% CI 3.8–9.8), *p* = 0.035) were significantly different (see Supplementary Material Figure S3).

**Figure 4:**
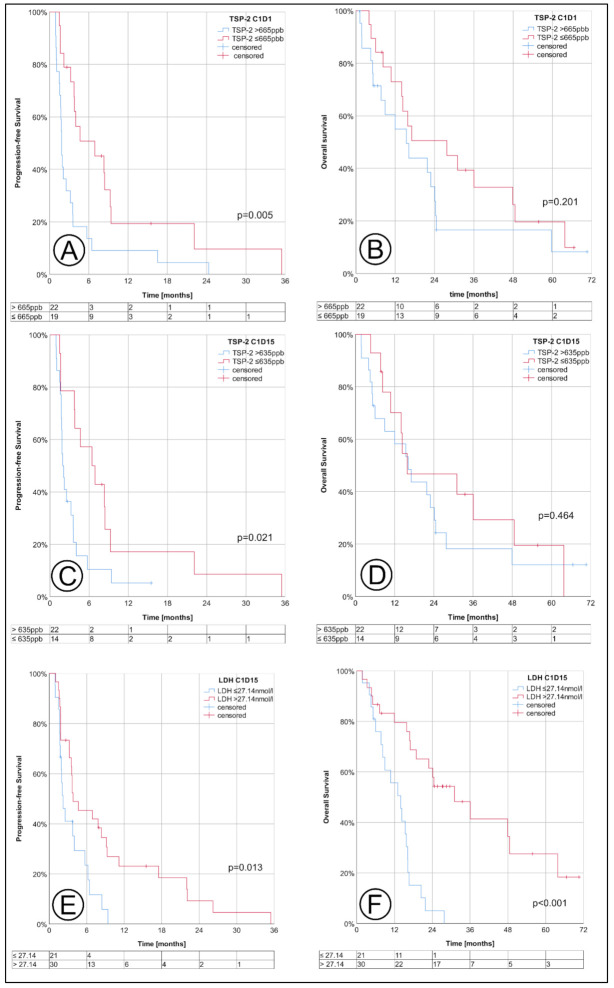
Progression-free survival (PFS) and overall survival (OS), stratified by thrombospondin-2 levels at baseline (see **A**,**B**), C1D15 (**C**,**D**) and by the lactate dehydrogenase level at C1D15 (**E**,**F**). The tables below each Kaplan–Meier diagram indicate the corresponding number of patients at risk; the analysis was performed in FAS (full analysis set).

Patients with a TSP-2 ≤665 ppb at C1D1 had a significantly longer median PFS (FAS: 6.9 months (95% CI 1.3–12.5) vs. 1.8 (95% CI 1.6–2.1), *p* = 0.005) both in the FAS and the PP cohort, but the OS did not significantly differ (see Table 1, Figure 4). Correspondingly, the 6-month PFS rate was higher for patients with a TSP-2 ≤665 ppb (50.8% (95% CI 26.8–70.7) vs. 13.6% (95% CI 3.4–30.9), *p* = 0.002). At C1D15, a TSP-2 ≤635 ppb identified patients with a significantly longer median PFS of 6.5 months (95% CI 2.3–10.5) vs. 2.0 (95% CI 1.6–2.3), *p* = 0.021). Patients with an LDH >27.14 nmol/L at C1D15 also had a longer median PFS (3.8 months (95% CI 0.4–7.3) vs. 2.2 (95% CI 1.4–3.0), *p* = 0.013) and a much longer median OS (31.0 months (95% CI 16.7–45.4) vs. 14.0 (95% CI 8.8–19.1), *p* < 0.001). In the landmark analysis, which set the landmark point to day 15, the cut-offs for TSP-2 at C1D1, C1D15, and LDH at C1D15 still separated responders from non-responders in terms of the PFS (all marker) and OS (only LDH C1D15) (see Supplementary Material Figure S4).

### 3.3. Predictive Impact of Biomarkers

TSP-2 at C1D1 had a significant association with disease progression in the multiple Cox regression, and patients with a TSP-2 ≤665 ppb had a significantly lower hazard ratio for progression (HR 0.36 (95% CI 0.16–0.76), *p* = 0.008, see Table 2). Patients ≥65 years (HR 0.31 (95% CI 0.14–0.66), *p* = 0.002) and patients with a BMI >25 kg/m² (HR 0.34 (95% CI 0.15–0.76), *p* = 0.008) also had a lower hazard ratio for progression in the multiple analysis. IMDC risk groups were only associated with the PFS in the univariate regression. The c-index of the cut-off for TSP-2 at C1D1 (665 ppb) to predict long therapy response was higher (0.76 (95% CI 0.56–0.97), *p* = 0.031) than the one for the pre-specified patient groups age (0.65 (95% CI 0.47–0.83), *p* = 0.12) and BMI (0.59 (95% CI 0.41–0.78), *p* = 0.31). As for the comparison of the biomarkers on day 15 with clinical characteristics, TSP-2 at C1D15 was again associated with the PFS in the multivariate regression analysis (HR 0.27 (95% CI 0.11–0.67), *p* = 0.004), besides BMI (HR 0.43 (95% CI 0.19–0.96), *p* = 0.039) and patient age (HR 0.25 (95% CI 0.11–0.58), *p* = 0.001, see Supplementary Material Table S3). The LDH at C1D15 and the IMDC risk groups were also associated with disease progression, but only in the univariate Cox regression analysis (see Supplementary Material Table S3).

Regarding overall survival, patients with a LDH >27.14 nmol/L at C1D15 (FAS: HR 0.21 (95% CI 0.09–0.48), *p* < 0.001) had a lower hazard ratio for death from any cause in the multiple Cox regression analysis, similar to patients with a BMI >25 kg/m² (HR 0.38 (95% CI 0.18–0.80), *p* = 0.011) or at poor risk according to IMDC (HR 3.36 (95% CI 1.37–8.22), *p* = 0.008, see Supplementary Material Tables S4 and S5). Neither TSP-2 nor other patient-related factors were associated with the OS in the multiple analysis. Within the landmark analysis, these results remained nearly unchanged and were still significant (see Supplementary Material Tables S6 and S7).

## 4. Discussion

For many cancer entities, such as breast (hormone receptor, Her2), colon (EGFR, BRAF, PI3K, PTEN, or NRAS mutation), or lung cancer (BRCA1/2 mutation, ALK/ ROS1 rearrangement), the utilization of predictive biomarkers became routine years ago and made personalized medicine a reality, but not for mRCC [11,12,13,14]. It is without doubt that new substance classes, especially checkpoint inhibitors and the latest combination of CI plus targeted therapy, have significantly improved therapy outcomes in mRCC in recent years [5]. However, a large proportion of patients still do not respond to therapy, but might profit from other therapeutic options instead. Hence, predictive biomarkers still represent an unmet need in mRCC therapy. Of note, predictive biomarkers have the potential to identify patients who are more likely to benefit from a specific drug, regardless of its overall proportion of therapy responders. Therefore, substances such as mTOR inhibitors, which are currently facing very low interest in the landscape of medical mRCC treatment as many patients do not respond, could return to clinical practice. Of note, reports about long therapy responders on everolimus clearly illustrate its high therapeutic potential—not for all, but some well-selected patients [15]. In this regard, the prospective MARC-2 trial searched for predictive blood biomarkers for patients with clear cell mRCC who would most likely benefit from treatment with everolimus after the failure of one VEGF-targeted therapy. Patients were enrolled from 2011 to 2015, when the only registered drugs in this setting were everolimus, axitinib, and sorafenib. The patient demographics and the clinical outcomes have been described elsewhere [7]. In addition to the clinical parameters, blood biomarkers as well as single-nucleotide polymorphisms (SNPs) in the mTOR gene were evaluated in this study. Our results confirmed that the baseline TSP-2 and LDH serum levels two weeks after therapy initiation had a significant association with therapy response and outcome. Both biomarkers had a greater predictive impact than the pre-specified patient or IMDC risk groups.

One major hallmark of the carcinogenesis of RCC is the uncontrolled upregulation of angiogenesis leading to an overproduction of pro-angiogenic factors, including VEGF or PDGF. mTOR inhibitors, such as everolimus, not only exert anti-angiogenic properties, but also inhibit the PI3-K/Akt/mTOR pathway, which is frequently overactivated in RCC. Hence, angiogenesis-related proteins not only appear to be excellent candidates for biomarkers for TKI, but also for mTOR inhibitors in mRCC, among which thrombospondins represent a family of multi-domain and multi-functional extracellular glycoproteins with five members, TSP-1 to TSP-5 [16]. In our analysis, non-responders to everolimus had higher serum levels of TSP-2 at baseline and after two weeks of treatment. We confirmed TSP-2 at baseline as an independent prognostic marker for the PFS. Compared to pre-specified patient groups stratified by BMI and age, which were also significant predictors for progression, the baseline TSP-2 had the highest c-index to discriminate long therapy responders from non-responders.

To the best of our knowledge, this is the first study illustrating a predictive value of soluble TSP-2 in (m)RCC so far. Boguslawska et al. analyzed the expression of THBS2, the gene encoding TSP-2, and found it to be significantly higher in RCCs compared to adjacent healthy kidney tissue. [17]. In contrast, an in vitro model of RCC demonstrated the anti-angiogenic properties of TSP-2, as the subcutaneous implantation of microbeads with TSP-2-producing cells significantly inhibited tumor growth and decreased the microvessel density [18]. Further contradictory conclusions about the role of TSP-2 have also been made for other tumor entities. While two studies demonstrated a potential diagnostic value for TSP-2 in pancreatic ductal adenocarcinoma, Nakamura et al. concluded that TSP-2 may act as a potent inhibitor of metastasis in pancreatic cancer [19,20,21]. In non-small-cell lung cancer, tumor patients had higher levels of TSP-2 in blood samples and primary tumor tissues compared to healthy controls, while the TSP-2 levels increased with advanced pT-stages [22,23]. In contrast, a low expression of TSP-2 was associated with advanced tumor or nodal stage and higher vascular invasion in colorectal cancer patients [24,25]. These divergent associations can be explained by the multiple functions of the TSP-2 protein, which interacts with various cytokines, proteases, cell-surface receptors, and growth factors [26]. Many authors clearly emphasize the angioinhibitory role of TSP-2 and its tumor-suppressive properties, but it rather appears to exert context-specific functions depending on the composition of the tumor microenvironment [27]. In our analysis, non-responders to everolimus had higher TSP-2 blood levels, a finding which requires further investigation in the future.

Other angiogenesis-related proteins, including PDGF-AA and angiopoietin-1 (ANG-1), also appeared to be deregulated between responders and non-responders within this study. However, the differences did not reach statistical significance. Recently, Lee et al. also found the blood levels of angiopoietin-2 to have predictive and prognostic properties in their biomarker analysis [28]. In another study comparing everolimus alone vs. lenvatinib alone vs. everolimus plus lenvatinib, angiogenesis- and inflammatory-related proteins, including angiopoietin-2 and IL-18BP, were also associated with the PFS or OS [29]. However, these results were only significant in the lenvatinib plus everolimus treatment arm. The RECORD-3 trial compared everolimus with sunitinib as the first but not second line of therapy for mRCC and the sequence of everolimus followed by sunitinib vs. the opposite regimen [30,31]. Therein, IL-18BP also predicted the first-line PFS of everolimus. Angiopoietin-1 and PDGF-B were included in the analysis, too, but they were not statistically significant. Unfortunately, our array analysis, which was mainly focused on angiogenesis-related proteins, did not include IL-18BP, which also represents another highly interesting predictive biomarker for everolimus, apart from thrombospondin-2.

As another major finding of our study, the LDH levels two weeks after therapy initiation identified therapy responders with a much longer overall survival. The prognostic value of LDH in RCC has frequently been hypothesized, and a meta-analysis confirmed a strong association of elevated LDH levels with a lower OS, irrespective of the histological subtype, and an association with the PFS [32]. Concerning a predictive role for LDH on mTOR inhibitors, Amato et al. analyzed 57 mRCC patients who received everolimus after prior TKI or interferon therapy. The baseline LDH was not only associated with the OS, but also the PFS [33]. Accordingly, Bodnar et al. found LDH to have a prognostic value in their prospective phase II trial with 58 patients who were treated with everolimus and had undergone previous treatments with one or two anti-angiogenic therapies. Patients with elevated LDH levels before treatment had a significantly shorter OS and PFS, but this difference was not significant [34]. However, one has to note that, within our study, only the LDH levels 14 days after therapy initiation had a significant and independent association with the OS and PFS. On the one hand, this can be interpreted as a limitation, as the known association of LDH and therapy response was not replicated by our data. On the other hand, LDH changes under therapy have not been analyzed for RCC so far. Instead, LDH kinetics have already been related to therapy response in patients with advanced non-small-cell lung cancer or recurrent metastatic nasopharyngeal carcinoma [35,36]. Moreover, a 1.5-fold increase in LDH over a period of three months has also been associated with an increased likelihood of a tumor relapse of diffuse large B-cell lymphomas [37]. In our analysis, the blood levels of LDH at C1D15 were higher in patients with a longer OS and PFS. To rule out the potential impact of a lead time bias, we performed a landmark analysis which confirmed these results. As the baseline LDH levels did not differ between the responders and non-responders, the increase in LDH in responders can be interpreted as therapy-related. This concept of the on-treatment monitoring of anti-tumor therapies is not new [38]. It is clear that on-treatment LDH levels cannot be interpreted as true prognostic or predictive biomarkers. However, patients with higher LDH levels 14 days after therapy initiation had a longer OS and PFS, which could help clinicians to decide whether or not to continue second-line everolimus therapy. In contrast, lower baseline TSP-2 levels were associated with longer therapy responses, which clearly has much more clinical impact as it could help to choose the right therapy.

However, everolimus no longer belongs to the current standard therapeutic landscape of mRCC therapy today and has been replaced by substances such as cabozantinib or nivolumab in the anti-VEGF-refractory setting. Against the background of long therapy responders and higher response rates in combination with lenvatinib, this has to be questioned [15,29]. The results of the CLEAR trial comparing lenvatinib + pembrolizumab vs. lenvatinib + everolimus vs. sunitinib have just recently proven the efficacy of the TKI + mTOR combination with a longer PFS compared to sunitinib, but it was shorter than with CI + TKI [39]. Moreover, everolimus + lenvatinib have also shown good results in the treatment of non-clear cell mRCC, further highlighting its therapeutic potential [40]. Therefore, everolimus should still be considered as a viable alternative in mRCC therapy, especially because our results might help clinicians to identify the right patients for everolimus-based therapy. Due to its involvement in angiogenesis, TSP-2 might also be tested for TKI-based regimens, potentially also including novel CI + TKI combinations, such as nivolumab + cabozantinib, which have recently proven excellent results [41]. Our findings also emphasize the high value of blood biomarkers in general, as liquid biopsies have the potential to present a minimally invasive snapshot of the current tumor situation at a defined time point [42]. In contrast, (primary) tumor tissue has often been retrieved years ago and does not reflect the genomic properties of tumor metastases [43,44]. For this reason, liquid biopsies appear to be a promising biomarker, because they may depict the evolution of tumor properties over time in a minimally invasive manner.

As another part of this exploratory prospective biomarker study, mTOR single-nucleotide polymorphisms were analyzed. More than 100 polymorphisms within the mTOR gene have been identified, and some of them have been linked with higher mTOR concentrations in RCC [45,46]. SNPs can also have prognostic implications, as carriers of some mTOR polymorphisms have a lower survival not only in RCC but also in other malignancies, such as esophageal cancer [47]. Within our analysis, four different mTOR SNPs were analyzed. Carriers of the CC genotype of rs2295080 had a tendency towards worse therapy response on day 56, indicating a predictive value. However, this was not statistically significant and is mainly related to an outlier within the CC group. Bodnar et al. also investigated polymorphisms in the mTOR (or FRAP1) gene and those encoding the PI3K/Akt/mTOR pathway. Carriers of the GG phenotype of rs2295080 also had a tendency to worse therapy response on everolimus, but again this was not significant [34]. In contrast, CC carriers compared to AC + AA carriers of the PIK3CA gene variant rs6443624 had a significantly longer PFS and OS [34]. In summary, further research is needed regarding the prognostic and predictive role of mTOR polymorphisms for mTOR therapy in mRCC.

This study is not devoid of limitations. Due to the lack of biomaterial, it was not possible to measure all potential biomarkers for all patients, limiting the statistical power. Furthermore, the total number of patients was limited and the known association of baseline LDH to patient survival could not be replicated. Moreover, this study has no implications for the predictive value of the proposed biomarkers TSP-2 and LDH in a therapeutic regimen including CIs. Nevertheless, we propose these two markers and potentially mTOR polymorphisms as well to be included in future trials with mTOR inhibitors to further test their predictive value.

## 5. Conclusions

In this prospective phase IV study analyzing the role of biomarkers in clear cell mRCC patients treated with everolimus, thrombospondin-2 and lactate dehydrogenase were found to have significant predictive power. mTOR polymorphisms had an impact on the therapy outcome with everolimus, but this finding was not significant. Hence, it appears to be feasible to identify patients showing longtime responses to everolimus and possible to increase the second-line response rates based on individual therapy selection. Possibly, our results might also be transferred to combination therapies including TKI or mTOR inhibitors. However, further research is needed in a randomized setting, also including CI, to reveal the impact of our findings.

## Figures and Tables

**Table 1:** Oncological outcome in the FAS (full analysis set), stratified by IMDC risk groups, thrombospondin-2 levels at baseline (C1D1) and C1D15, and the lactate dehydrogenase level at C1D15.

Variable	Subgroup	Patients	6-Month PFS (%) (95%CI)	Median PFS (Months) (95%CI)	Median OS (Months) (95%CI)
all patients	-	63	39.3 (27.0–31.5)	3.8 (3.2–6.2)	16.8 (14.3–24.3)
IMDC risk groups	favorable	7	47.6 (7.5–80.8)	3.7 (0–8.4)	20.4 (NA–NA)
	intermediate	40	44.6 (28.9–59.2)	5.3 (1.6–8.9)	18.9 (9.9–27.9)
	poor	11	9.1 (0.5–33.3)	3.6 (1.6–5.5)	6.8 (3.8–9.8)
TSP-2 C1D1	≤665 ppb	19	50.8 (26.8–70.7)	6.9 (1.3–12.5)	27.8 (3.3–52.2)
	>665 ppb	22	13.6 (3.4–30.9)	1.8 (1.6–2.1)	15.4 (5.6–25.2)
TSP-2 C1D15	≤635 ppb	14	57.1 (28.4–78.0)	6.5 (2.3–10.5)	15.8 (0.0–35.6)
	>635 ppb	22	10.4 (1.8–27.6)	2.0 (1.6–2.3)	16.2 (8.8–23.6)
LDH C1D15	≤27.14 nmol/L	21	17.6 (4.5–37.7)	2.2 (1.4–3.0)	14.0 (8.8–19.1)
	>27.14 nmol/L	30	41.9 (24.1–58.8)	3.8 (0.4–7.3)	31.0 (16.7–45.4)

95% CI: 95% confidence interval; IMDC: International Metastatic Renal Cell Carcinoma Database Consortium; PFS: Progression-free survival; OS: Overall survival; TSP-2: Thrombospondin 2; LDH: Lactate dehydrogenase.

**Table 2:** Univariate and multiple Cox regression analysis to compare the impact of pre-specified patient groups and the exploratory biomarkers at baseline on the progression-free survival. The number next to each group indicates the quantity of patients included within the corresponding analysis.

Variable	Subgroup	Univariate			Multiple		
		Pat.	HR (95%CI)	*p*-Value	Pat.	HR (95%CI)	*p*-Value
age	<65 years	31	1	0.004	18	1	0.002
	≥65 years	32	0.45 (0.26–0.78)		20	0.31 (0.14–0.66)	
gender	male	48	-	0.149	-	-	-
	female	15	-		-	-	
BMI	≤25 kg/m²	22	1	0.042	12	1	0.008
	>25 kg/m²	41	0.57 (0.33–0.98)		26	0.34 (0.15–0.76)	
IMDC risk groups	fav.+ interm.	47	1	0.029	30	-	0.779
	poor	11	2.16 (1.08–4.3)		8	-	
TSP-2 C1D1	>665 ppb	22	1	0.007	19	1	0.008
	≤665 ppb	19	0.40 (0.20–0.78)		19	0.36 (0.16–0.76)	

95% CI: 95% confidence interval; BMI: Body mass index; HR: Hazard ratio; IMDC: International Metastatic Renal Cell Carcinoma Database Consortium TSP-2: Thrombospondin 2; pat.: Patients.

## Data Availability

The data that support the findings of this study are available from the corresponding author upon reasonable request.

## Supplementary Materials

The following are available online at https://www.mdpi.com/article/10.3390/cancers13112594/s1. Table S1: Absolute genotype distribution of the mTOR polymorphisms mTOR3162/rs2295080, mTOR3099/rs2295079 and mTOR8600/rs2536, mTOR8167/rs12139042 in 54 patients. Table S2: Overall response at day 56, stratified by the mTOR3162/rs2295080 polymorphism. Table S3: Univariate and multiple Cox regression analysis to compare the impact of pre-specified patient groups and the exploratory biomarkers on day 15 on the progression-free survival. The number next to each group indicates the quantity of patients included within the corresponding analysis. Table S4: Univariate and multiple Cox Regression analysis to compare the impact of pre-specified patient groups and the baseline exploratory biomarkers on the overall survival in the full-analysis set (FAS). The number next to each group indicates the quantity of patients included within the corresponding analysis. Table S5: Univariate and multiple Cox Regression analysis to compare the impact of pre-specified patient groups and the exploratory biomarkers on day 15 on the overall survival in the full-analysis set (FAS). The number next to each group indicates the quantity of patients included within the corresponding analysis. Table S6: To rule out the impact of a potential lead time bias on the results, the Cox regression analysis for the progression-free survival was repeated for the biomarkers on day 15, with the landmark set to day 15. Table S7: Cox Regression analysis to compare the impact of pre-specified patient groups and the exploratory biomarkers on day 15 on the overall survival in the full-analysis set, with the landmark set to day 15. Figure S1: Angiopoietin-1, the platelet-derived-growth factor-AA (PDGF-AA) and thrombospondin-2 (TSP-2) appeared to increase in non-responders, but to decrease in long responders in the screening cohort comprising 12 patients (eight responders, four non-responders), measured by antibody array. Figure S2: ROC analyses to calculate the optimal cut-offs for baseline Thrombospondin-2 (A), thrombospondin-2 at day 15 (B) and LDH at day 15 (C) to discriminate long-term responders from early non-responders. Figure S3: Progression-free survival (A,C) and overall survival (B,D), stratified by IMDC risk groups in the FAS. For C and D, patients at favorable and intermediate risk were compared with poor risk. Figure S4: As a part of the landmark analysis, the Kaplan Meier analyses for progression-free survival (PFS) and overall survival (OS), stratified by TSP-2 levels at baseline (see A,B), C1D15 (see C,D) and by LDH level at C1D15 (see E,F), were calculated again with the landmark set to day 15 in order to determine the impact of a potential lead time bias.
