# Supplementary material for: Thrombospondin-2 and LDH Are Putative Predictive Biomarkers for Treatment with Everolimus in Second-Line Metastatic Clear Cell Renal Cell Carcinoma (MARC-2 Study)
Source: Cancers (Basel). 2021 May 25;13(11):2594. doi: 10.3390/cancers13112594 (PMC8199288; doi:10.3390/cancers13112594)
Supplement: Supplementary file 1 [file cancers-13-02594-s001.zip › cancers-1197045-supplementary.pdf]

cancers

MDPI

Article

Thrombospondin-2 and LDH Are Putative Predictive Biomarkers for Treatment with Everolimus in Second-Line Metastatic Clear Cell Renal Cell Carcinoma (MARC-2 Study)

Philip Zeuschner, Sebastian Hölters, Michael Stöckle, Barbara Seliger, Anja Mueller, Hagen S. Bachmann, Viktor Grünwald, Daniel C. Christoph, Arnulf Stenzl, Marc-Oliver Grimm, Fabian Brüning, Peter J. Goebell, Marinela Augustin, Frederik Roos, Johanna Harde, Iris Benz-Rüd, Michael Staehler and Kerstin Junker

Supplementary Materials

Table S1. Absolute genotype distribution of the mTOR polymorphisms mTOR3162/rs2295080, mTOR3099/rs2295079 and mTOR8600/rs2536, mTOR8167/rs12139042 in 54 patients. As mTOR3162/mTOR3099 and mTOR8600/mTOR8167 always have the corresponding genotype, only the alleles of mTOR3162 and mTOR8600 are shown.

		rs2536/rs12139042		
		**AA**	**AG**	**Total**
rs2295080/rs2295079	**AA**	23	0	23
	**AC**	17	5	22
	**CC**	7	2	9
Total		47	7	54

Table S2. Overall response at day 56, stratified by the mTOR3162/rs2295080 polymorphism.

	**AA**	**AC**	**CC**
**Partial response (PR)**	1 (5%)	-	1 (14%)
**Stable disease (SD)**	12 (60%)	15 (71%)	3 (43%)
**Progressive disease (PD)**	7 (35%)	6 (29%)	3 (43%)
Total	20 (100%)	21 (100%)	7 (100%)

Table S3. Univariate and multiple Cox Regression analysis to compare the impact of pre-specified patient groups and the exploratory biomarkers on day 15 on the progression-free survival in the full-analysis set (FAS). The number next to each group indicates the quantity of patients included within the corresponding analysis.

		**Univariate**			**Multiple**		
		**Pat.**	**HR (95%CI)**	***p*-Value**	**Pat.**	**HR (95%CI)**	***p*-Value**
**Age**	<65 years	31	1	0.004	16	1	0.001
	≥65 years	32	0.45 (0.26–0.78)		18	0.25 (0.11–0.58)	
**Gender**	male	48	-	0.082	-	-	-
	female	15	-		-	-	
**BMI**	≤25kg/m²	22	1	0.042	11	1	0.039
	>25 kg/m²	41	0.57 (0.33–0.98)		23	0.43 (0.19–0.96)	
**IMDC Risk Groups**	fav. + interm.	47	1	0.029	28	-	0.565
	poor	11	2.16 (1.08–4.3)		6	-	
**TSP-2 C1D15**	>635 ppb	22	1	0.025	20	1	0.004
	≤635 ppb	14	0.42 (0.19–0.90)		14	0.27 (0.11–0.67)	
**LDH C1D15**	≤27.14 nmol/L	21	1	0.015	15	-	0.188
	>27.14 nmol/L	30	0.46 (0.24–0.86)		19	-	

95%CI: 95% confidence interval, BMI: body mass index, HR: hazard ratio, IMDC: International Metastatic Renal Cell Carcinoma Database Consortium, LDH: lactate dehydrogenase, pat.: patients, TSP-2: thrombospondin 2.

Table S4. Univariate and multiple Cox Regression analysis to compare the impact of pre-specified patient groups and the baseline exploratory biomarkers on the overall survival in the full-analysis set (FAS). The number next to each group indicates the quantity of patients included within the corresponding analysis.

		Univariate			Multiple		
		**Pat.**	**HR (95%CI)**	***p*-Value**	**Pat.**	**HR (95%CI)**	***p*-Value**
**Age**	<65 years	31	-	0.226	-	-	-
	≥65 years	32	-		-	-	
**Gender**	male	48	-	0.082	-	-	-
	female	15	-		-	-	
**BMI**	≤25 kg/m²	22	1	0.010	21	1	<0.001
	>25 kg/m²	41	0.45 (0.25–0.83)		37	0.29 (0.15–0.56)	
**IMDC Risk Groups**	fav. + interm.	47	1	0.039	47	1	0.015
	poor	11	2.14 (1.04–4.40)		11	2.5 (1.19–5.31)	
**TSP-2 C1D1**	>665 ppb	22	-	0.201	-	-	-
	≤665 ppb	19	-		-	-	

95%CI: 95% confidence interval, BMI: body mass index, HR: hazard ratio, IMDC: International Metastatic Renal Cell Carcinoma Database Consortium, pat.: patients, TSP-2: thrombospondin 2.

Table S5. Univariate and multiple Cox Regression analysis to compare the impact of pre-specified patient groups and the exploratory biomarkers on day 15 on the overall survival in the full-analysis set (FAS). The number next to each group indicates the quantity of patients included within the corresponding analysis.

		Univariate			Multiple		
		**Pat.**	**HR (95%CI)**	***p*-Value**	**Pat.**	**HR (95%CI)**	***p*-Value**
**Age**	<65 years	31	-	0.226	-	-	-
	≥65 years	32	-		-	-	
**Gender**	male	48	-	0.082	-	-	-
	female	15	-		-	-	
**BMI**	≤25 kg/m²	22	1	0.010	16	1	0.011
	>25 kg/m²	41	0.45 (0.25–0.83)		31	0.38 (0.18–0.80)	
**IMDC Risk Groups**	fav. + interm.	47	1	0.039	39	1	0.008
	poor	11	2.14 (1.04–4.40)		8	3.36 (1.37–8.22)	
**TSP-2 C1D15**	>635 ppb	22	-	0.465	-	-	-
	≤635 ppb	14	-		-	-	
**LDH C1D15**	≤27.14 nmol/L	21	1	<0.001	18	1	<0.001
	>27.14 nmol/L	30	0.20 (0.09–0.43)		29	0.21 (0.09–0.48)	

95%CI: 95% confidence interval, BMI: body mass index, HR: hazard ratio, IMDC: International Metastatic Renal Cell Carcinoma Database Consortium, LDH: lactate dehydrogenase, pat.: patients, TSP-2: thrombospondin 2.

Table S6. To rule out the impact of a potential lead time bias on the results, the Cox regression analysis for the progression-free survival was repeated for the biomarkers on day 15, with the landmark set to day 15.

		Univariate			Multiple		
		**Pat.**	**HR (95%CI)**	***p*-Value**	**Pat.**	**HR (95%CI)**	***p*-Value**
**Age**	<65 years	31	1	0.005	16	1	0.001
	≥65 years	32	0.46 (0.27–0.79)		18	0.25 (0.11–0.58)	
**Gender**	male	48	-	0.166	-	-	-
	female	15	-		-	-	
**BMI**	≤25 kg/m²	22	1	0.036	11	1	0.040
	>25 kg/m²	41	0.56 (0.32–0.96)		23	0.43 (0.19–0.96)	
**IMDC Risk Groups**	fav. + interm.	47	1	0.023	28	-	0.568
	poor	11	2.22 (1.12–4.42)		6	-	
**TSP-2 C1D15**	>635 ppb	22	1	0.025	20	1	0.005
	≤635 ppb	14	0.42 (0.19–0.90)		14	0.27 (0.11–0.67)	
**LDH C1D15**	≤27.14 nmol/L	21	1	0.017	15	-	0.189
	>27.14 nmol/L	30	0.46 (0.24–0.87)		19	-	

95%CI: 95% confidence interval, BMI: body mass index, HR: hazard ratio, IMDC: International Metastatic Renal Cell Carcinoma Database Consortium, LDH: lactate dehydrogenase, pat.: patients, TSP-2: thrombospondin 2.

Table S7. Cox Regression analysis to compare the impact of pre-specified patient groups and the exploratory biomarkers on day 15 on the overall survival in the full-analysis set, with the landmark set to day 15.

		Univariate			Multiple		
		**Pat.**	**HR (95%CI)**	***p*-Value**	**Pat.**	**HR (95%CI)**	**p-value**
**Age**	<65 years	31	-	0.226	-	-	-
	≥65 years	32	-		-	-	
**Gender**	male	48	-	0.079	-	-	-
	female	15	-		-	-	
**BMI**	≤25 kg/m²	22	1	0.011	16	1	0.013
	>25 kg/m²	41	0.45 (0.25–0.83)		31	0.38 (0.18–0.82)	
**IMDC Risk Groups**	fav. + interm.	47	1	0.039	39	1	0.008
	poor	11	2.14 (1.04–4.41)		8	3.35 (1.34–8.19)	
**TSP-2 C1D15**	>635 ppb	22	-	0.465	-	-	-
	≤635 ppb	14	-		-	-	
**LDH C1D15**	≤27.14 nmol/L	21	1	<0.001	18	1	<0.001
	>27.14 nmol/L	30	0.20 (0.10–0.43)		29	0.22 (0.09–0.49)	

95%CI: 95% confidence interval, BMI: body mass index, HR: hazard ratio, IMDC: International Metastatic Renal Cell Carcinoma Database Consortium, LDH: lactate dehydrogenase, pat.: patients, TSP-2: thrombospondin 2.

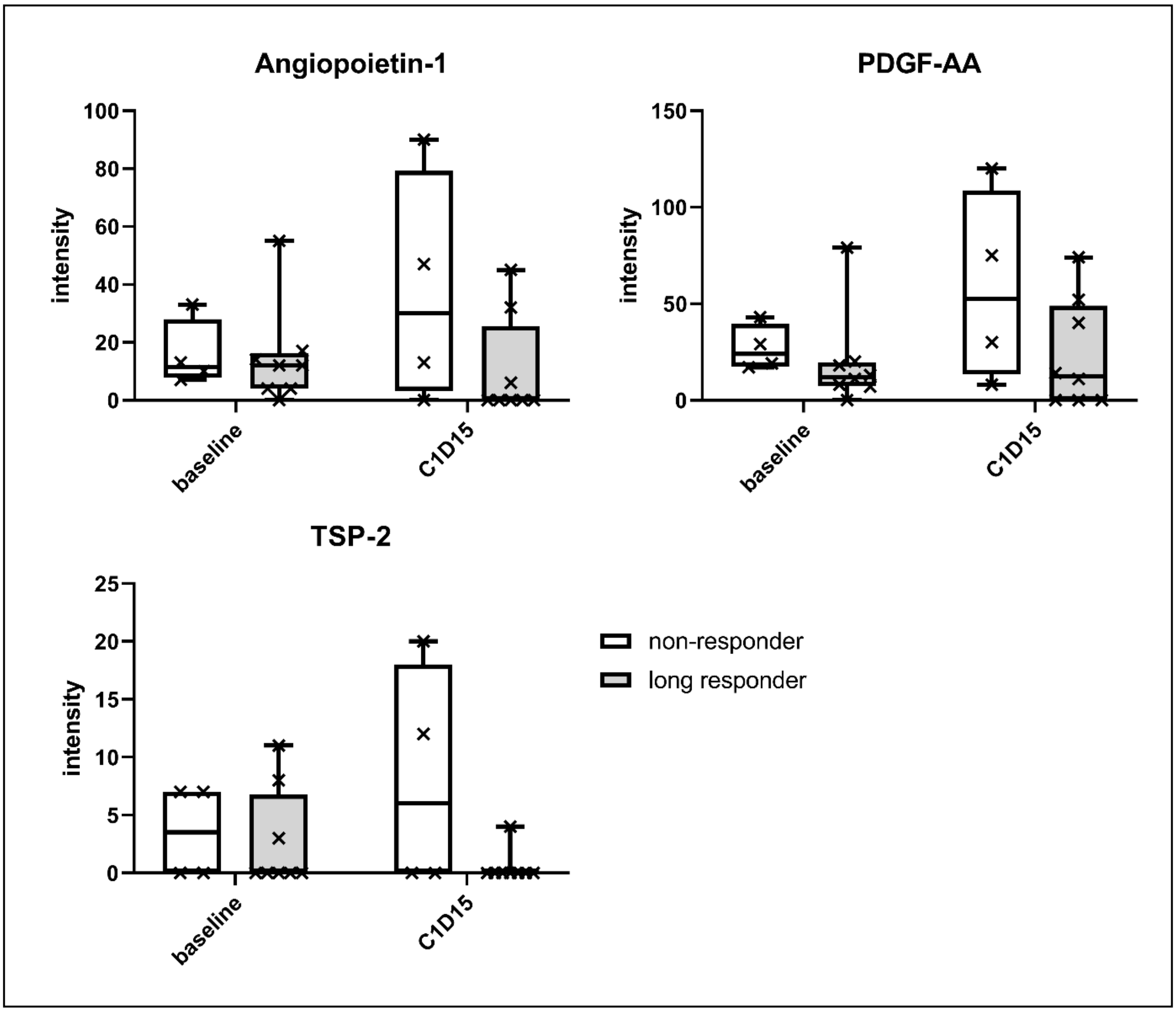

Figure S1. Angiopoietin-1, the platelet-derived-growth factor-AA (PDGF-AA) and thrombospondin-2 (TSP-2) appeared to increase in non-responders, but to decrease in long responders in the screening cohort comprising 12 patients (eight responders, four non-responders), measured by antibody array.

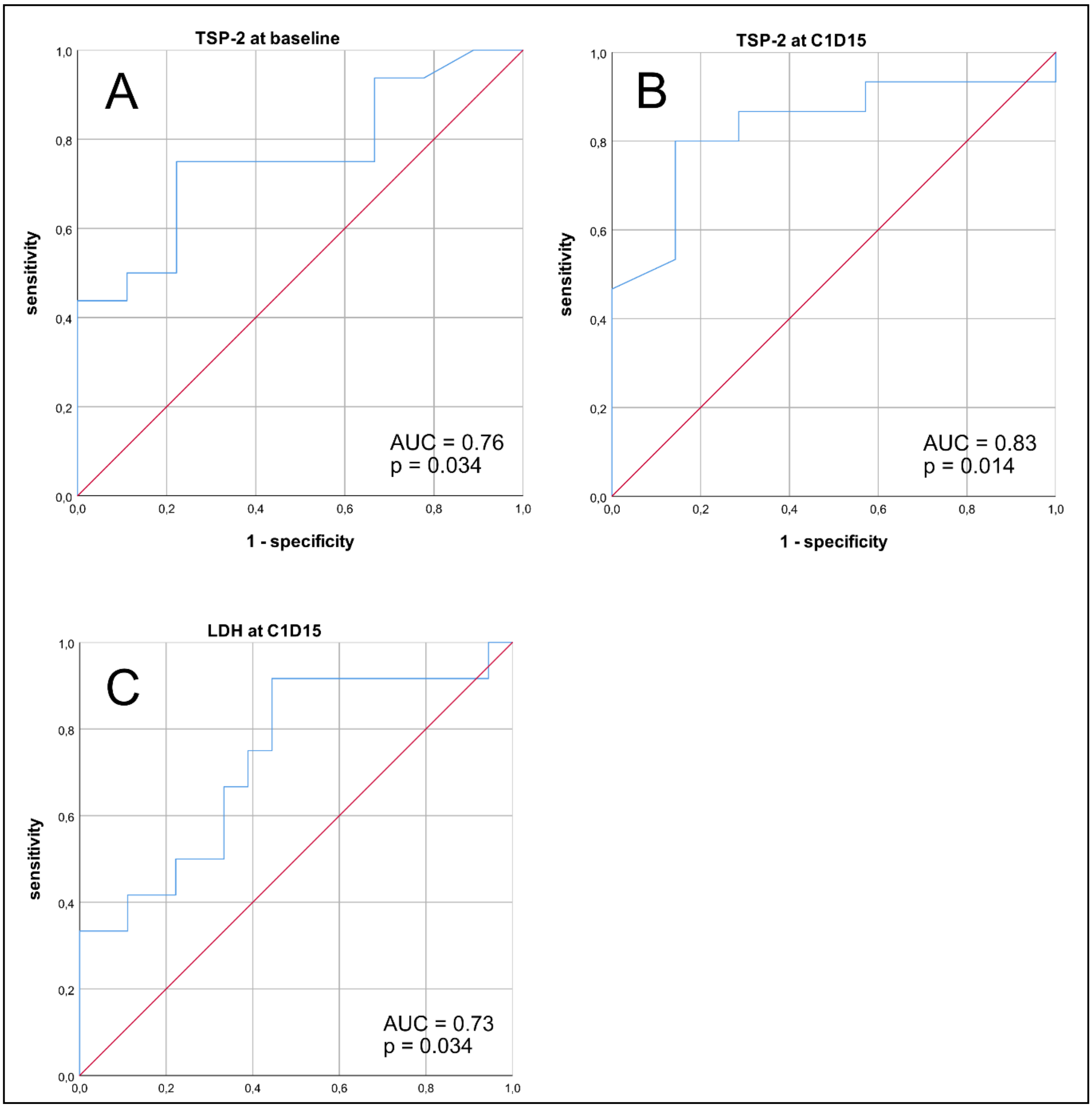

Figure S2. ROC analyses to calculate the optimal cut-offs for baseline Thrombospondin-2 (**A**), thrombospondin-2 at day 15 (**B**) and LDH at day 15 (**C**) to discriminate long-term responders from early non-responders.

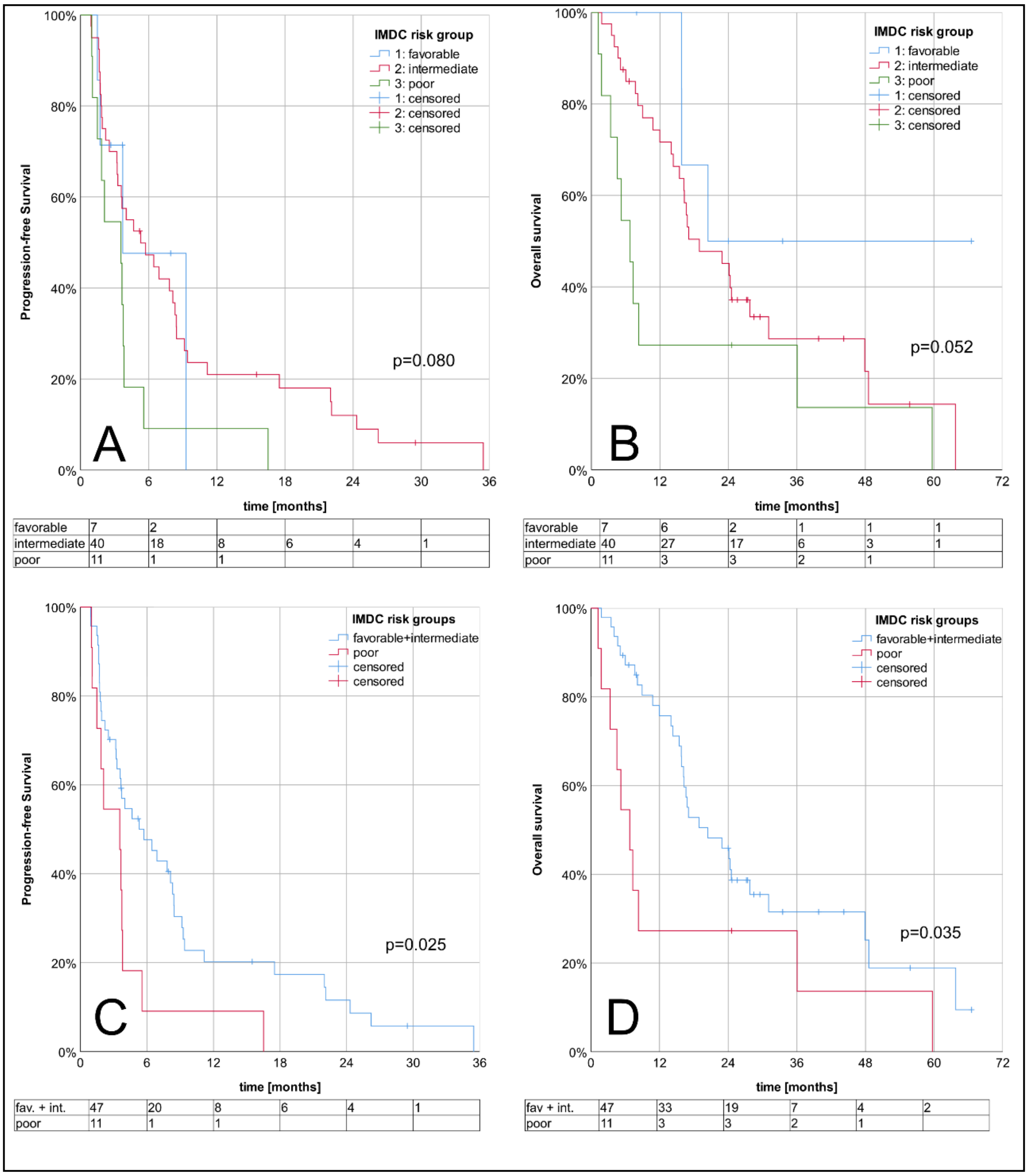

A (time [months]: 0, 6, 12, 18, 24, 30)

	0	6	12	18	24	30
favorable	7	2				
intermediate	40	18	8	6	4	1
poor	11	1	1			

B (time [months]: 0, 12, 24, 36, 48, 60)

	0	12	24	36	48	60
favorable	7	6	2	1	1	1
intermediate	40	27	17	6	3	1
poor	11	3	3	2	1	

C (time [months]: 0, 6, 12, 18, 24, 30)

	0	6	12	18	24	30
fav. + int.	47	20	8	6	4	1
poor	11	1	1			

D (time [months]: 0, 12, 24, 36, 48, 60)

	0	12	24	36	48	60
fav + int.	47	33	19	7	4	2
poor	11	3	3	2	1	

Figure S3. Progression-free survival (**A**,**C**) and overall survival (**B**,**D**), stratified by IMDC risk groups in the FAS. For (**C**) and (**D**), patients at favorable and intermediate risk were compared with poor risk. The tables below each Kaplan Meier diagram indicate the corresponding number of patients at risk.

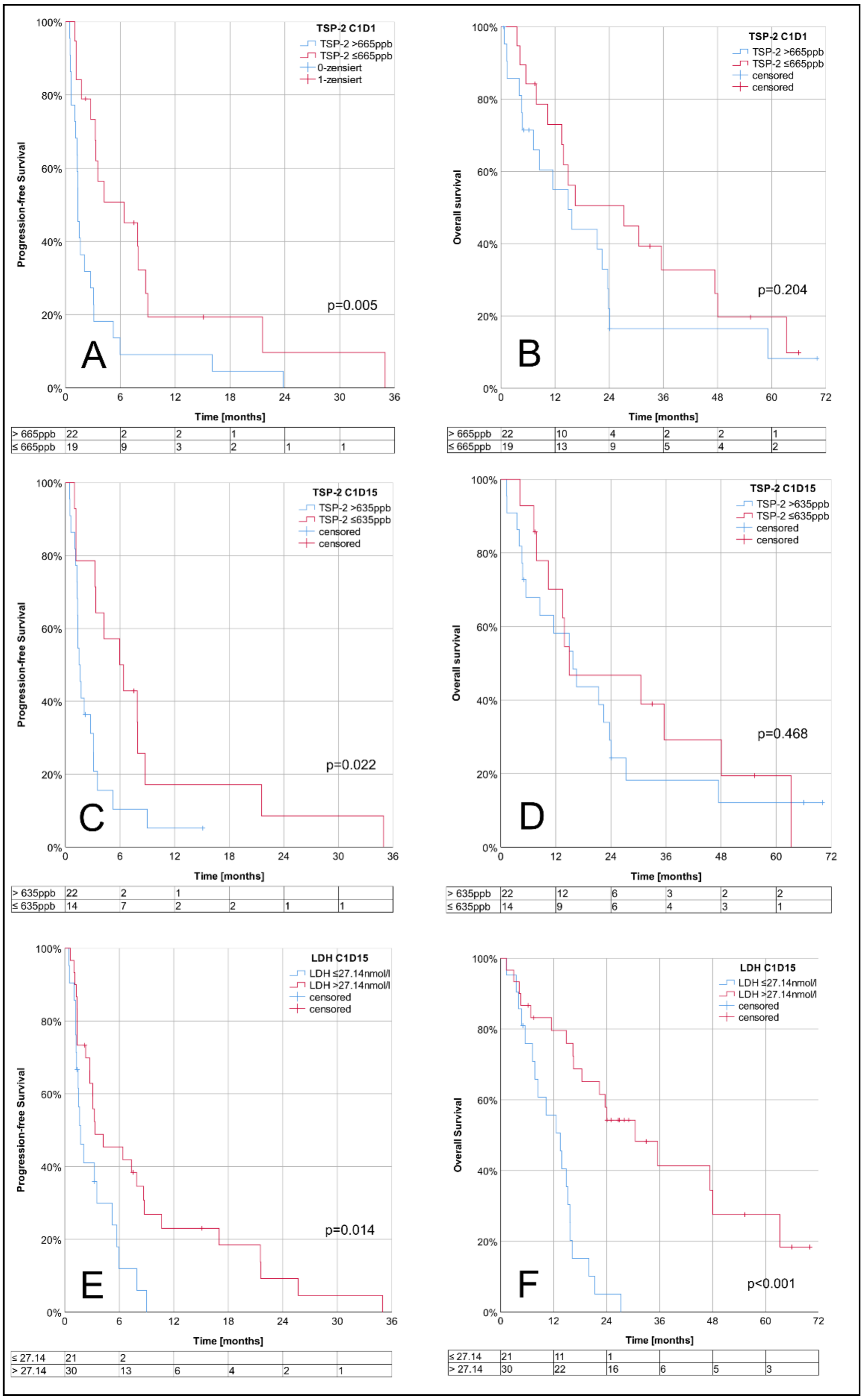

Figure S4. As a part of the landmark analysis, the Kaplan Meier analyses for progression-free survival (PFS) and overall survival (OS), stratified by TSP-2 levels at baseline, see (**A**,**B**), C1D15 (**C**,**D**) and by LDH level at C1D15, were calculated again with the landmark set to day 15 in order to determine the impact of a potential lead time bias.
